# Immunological aspects of *Giardia* infections

**DOI:** 10.1051/parasite/2014056

**Published:** 2014-10-28

**Authors:** Martin F. Heyworth

**Affiliations:** 1 Research Service, Department of Veterans Affairs (VA) Medical Center Philadelphia PA 19104 USA; 2 Department of Medicine, Perelman School of Medicine, University of Pennsylvania Philadelphia PA 19104 USA

**Keywords:** *Giardia intestinalis*, Giardiasis, NO

## Abstract

Immunodeficiency, particularly antibody deficiency, predisposes to increased intensity and persistence of *Giardia* infections. *Giardia*-infected immunocompetent hosts produce serum and intestinal antibodies against *Giardia* trophozoites. The number of *Giardia muris* trophozoites, in mice with *G. muris* infection, is reduced by intra-duodenal administration of anti-*G. muris* antibody. *Giardia intestinalis* antigens that are recognised by human anti-trophozoite antibodies include variable (variant-specific) and invariant proteins. Nitric oxide (NO) appears to contribute to host clearance of *Giardia* trophozoites. Arginine is a precursor of NO and is metabolised by *Giardia* trophozoites, possibly reducing its availability for generation of NO by the host. Work with mice suggests that T lymphocytes and interleukin-6 (IL-6) contribute to clearance of *Giardia* infection via mechanisms independent of antibodies.

## Abstract (fr)

L’immunodéficience, particulièrement la déficience en anticorps, prédispose à une augmentation de l’intensité et de la durée des infections à *Giardia*. Les hôtes immunocompétents infectés par *Giardia* produisent des anticorps sériques et intestinaux contre les trophozoïtes de *Giardia*. Le nombre de trophozoïtes de *Giardia muris*, chez la souris infectée par *G. muris*, est réduit par l’administration intra-duodénale d’anticorps anti *G. muris*. Les antigènes de *Giardia intestinalis* qui sont reconnus par des anticorps humains anti-trophozoïtes comprennent des protéines variables (spécifiques aux variants) et des protéines invariantes. L’oxyde nitrique (NO) semble contribuer à débarrasser l’hôte des trophozoïtes de *Giardia*. L’arginine est un précurseur de NO et est métabolisé par les trophozoïtes de *Giardia*, ce qui réduit peut-être sa disponibilité pour la production de NO par l’hôte. Les travaux sur la souris suggèrent que les lymphocytes T et l’interleukine-6 (IL-6) contribuent à éliminer *Giardia* par des mécanismes indépendants des anticorps.

*Giardia intestinalis* (synonyms: *G. duodenalis*, *G. lamblia*) is a protozoan parasite that colonises the small intestinal lumen of vertebrate hosts. In human subjects, *G. intestinalis* infections range in clinical severity from asymptomatic colonisation to a debilitating syndrome that includes chronic diarrhoea and malabsorption. The two-stage life cycle of *Giardia* species comprises the motile trophozoite, with eight flagella and a ventral adhesive disc by which it adheres to the luminal surface of intestinal epithelial cells (and thereby resists peristaltic expulsion from the host’s intestine), and the thick-walled cyst, which is excreted from the host. Previously uninfected hosts become infected by oral ingestion of *Giardia* cysts.

*Giardia* infections are increased in intensity and/or duration in human or non-human mammalian hosts with various forms of immunodeficiency, in comparison with their immunocompetent counterparts [9, 14, 20]. This situation indicates that host immunological responses limit the intensity and/or duration of these infections. The extant literature suggests that impaired production of anti-*Giardia* antibodies is the main reason why immunodeficiency states predispose to severe/prolonged *Giardia* infections [4]. From the 1980s onwards, it has been known that *Giardia*-infected human and non-human hosts generate serum and intestinal antibody responses against *Giardia* trophozoites [10, 28]. Anti-*Giardia* IgA is present in the intestinal lumen of *Giardia*-infected hosts and has also been detected in human milk [10, 32]. Colostrum from cows with *G. intestinalis*-infected calves contains anti-*Giardia* IgG [19]. Intraperitoneal or intraduodenal administration of anti-*G. muris* antibody leads to reduction in the number of intestinal *G. muris* trophozoites, in mice infected with this parasite [1, 3]. This result is consistent with a role for antibodies in clearing *G. muris* from the mouse intestinal lumen.

*Giardia* trophozoite antigens that are recognised by antibodies of *Giardia*-infected hosts include heterogeneous “variant-specific surface proteins” (VSPs), and non-variable (structurally conserved) proteins [21]. Of a repertoire of approximately 150 (or more) VSPs in *Giardia intestinalis*, only one VSP appears to be expressed on an individual *Giardia* trophozoite at any one time, other than during antigenic “switching” [17, 18]. It has been speculated that antigenic switching by *Giardia* trophozoites, whereby expression of one VSP changes to that of a different VSP, might be an immune evasion strategy (an adaptation by the parasite to the presence of host antibodies directed against whichever VSP is initially expressed by a population of trophozoites in the intestinal lumen) [17]. The observation that *G. intestinalis* trophozoites switch from the expression of one VSP to another in the absence of antibodies, during *in vitro* culture [18], does not rule out the possibility that antibodies might select against the persistence of initially expressed VSP(s) in the host. The biological role, if any, of VSPs appears to be unknown, although it has been postulated that expression of a particular VSP might influence the relative ability of *Giardia* trophozoites to colonise a particular species of host [26]. *Giardia* trophozoites genetically engineered to express “numerous” VSPs simultaneously can act as a vaccine (whether given as live organisms, or as an inanimate mixture of antigens) to generate protective anti-*Giardia* immunity in a gerbil host [24]. The implications of this finding for understanding the “normal” mechanism(s) of host protective immunity against *Giardia* infection(s) are, however, unclear.

Sera from *G. intestinalis*-infected human subjects contain antibodies directed against trophozoite VSPs [23]. Of possibly greater biological significance, antibodies against *G. intestinalis* trophozoite proteins that are structurally conserved (invariant) have also been identified in sera from *Giardia*-infected individuals. Trophozoite invariant proteins recognised by human serum antibodies include *α*-giardins (a group of proteins originally regarded as “internal” in trophozoite adhesive discs, though later identified on trophozoite surface membranes and flagella) [22, 33–35], fructose-1,6-bisphosphate aldolase, and *G. intestinalis* enzymes involved in arginine metabolism (arginine deiminase and ornithine carbamoyl transferase) [21]. Host immunological memory is suggested by the isolation of *Giardia*-reactive CD4+ T lymphocytes from the peripheral blood of human subjects known to have been infected with *G. intestinalis* during an outbreak of giardiasis 5 years previously [7]. One can speculate that these CD4+ T lymphocytes included cells that were able to provide “help” for *Giardia*-specific antibody production by B lymphocytes.

A plausible mechanism for presumed antibody-mediated clearance of *Giardia* infections would involve prevention (by antibodies) of trophozoite attachment to the host intestinal epithelium [8] followed by peristaltic expulsion of these organisms from the intestine. Reportedly, an antibody against *δ*-giardin (a protein in trophozoite adhesive discs) inhibits *G. intestinalis* trophozoite attachment to non-biological surfaces; however, the pertinent antibody may (also) have killed trophozoites, as judged by their morphology after exposure to the antibody [12]. Oral administration of a *Salmonella enterica* strain bioengineered to express *G. intestinalis α*-1 giardin induced serum IgG and intestinal IgA antibodies against *α*-1 giardin in mice, and conferred some protection against subsequent *G. intestinalis* infection in the animals [11]. Although recombinant *α*-1 giardin of *G. intestinalis* binds to human intestinal epithelial cells *in vitro* [34], exposure of *G. intestinalis* trophozoites to antibody directed against α-1 giardin did not inhibit the ability of these organisms to become attached to a non-biological surface [6]. Further work may be needed to clarify the role, if any, of antibodies against giardin(s) in clearance of/protection against *Giardia* infections.

Experimental work with mice has suggested that T lymphocytes can contribute directly (i.e., in the absence of antibodies) to clearance of infection with a clone of *G. intestinalis* (GS/M-H7) [27]. The mechanism(s) involved in this putative T-cell-mediated clearance of *Giardia* infection does not appear to be known (it may be worth mentioning that a postulated effector role for T cells in the clearance process would not be identical to CD4+ T-cell-mediated help for anti-*Giardia* antibody production) [27].

Studies of *Giardia* infections in rodents have implicated interleukin-6 (IL-6) in anti-*Giardia* immunity. IL-6-deficient mice have a diminished ability to clear infection caused by *G. intestinalis* [2, 36]. The mice studied in the pertinent experiments were able to produce intestinal anti-trophozoite IgA; the findings suggest that IL-6 contributes to clearance of *Giardia* infection in mice (albeit by an unknown mechanism that appears not to involve IgA). Recent work has identified dendritic cells (of bone marrow origin) as a source of IL-6 that promotes clearance of *G. intestinalis* infection in mice [13].

There is evidence that intestinal nitric oxide (NO) contributes to host clearance of *Giardia* trophozoites [5]. In view of the fact that arginine is a substrate for generation of NO, it is interesting that *Giardia* trophozoites appear to compete with the host for arginine [5]. *Giardia* trophozoites are able to metabolise arginine [25]. Uptake and metabolism of arginine by *Giardia* trophozoites has implications for host nutrition (reducing the proportion of dietary arginine available for absorption by the host), as well as for trophozoite survival via reduced availability of arginine for host NO production [29, 30].

Experimental work has implicated mast cells in clearance of *Giardia* infections, at least in mice [16]. There is evidence of increased expression of mast cell protease genes during *Giardia* infections in mice [31], though whether this increased gene expression contributes to a possible role for mast cells in promoting intestinal peristalsis (and consequent expulsion of *Giardia* trophozoites from the intestinal lumen) is unclear [16].

As mentioned above, mice can be partially protected against *Giardia* infection, by oral administration of bacteria that have been genetically engineered to express *Giardia* proteins [11, 15]. There would seem to be little clinical imperative to try to develop a vaccine against human giardiasis, even if doing so were eventually found to be technically feasible.

*Author’s Disclaimer*: The content of this article does not necessarily reflect the views of the US Department of Veterans Affairs or of the US Government.

## References

[1] Belosevic M, Faubert GM, Dharampaul S. 1994. Antimicrobial action of antibodies against *Giardia muris* trophozoites. Clinical and Experimental Immunology, 95, 485–489 8137543 10.1111/j.1365-2249.1994.tb07023.x PMC1535076

[2] Bienz M, Dai WJ, Welle M, Gottstein B, Mȕller N. 2003. Interleukin-6-deficient mice are highly susceptible to *Giardia lamblia* infections but exhibit normal intestinal immunoglobulin A responses against the parasite. Infection and Immunity, 71, 1569–1573 12595479 10.1128/IAI.71.3.1569-1573.2003 PMC148820

[3] Butscher WG, Faubert GM. 1988. The therapeutic action of monoclonal antibodies against a surface glycoprotein of *Giardia muris*. Immunology, 64, 175–180 2454885 PMC1385204

[4] Eckmann L. 2003. Mucosal defences against *Giardia*. Parasite Immunology, 25, 259–270 12969444 10.1046/j.1365-3024.2003.00634.x

[5] Eckmann L, Laurent F, Langford TD, Hetsko ML, Smith JR, Kagnoff MF, Gillin FD. 2000. Nitric oxide production by human intestinal epithelial cells and competition for arginine as potential determinants of host defense against the lumen-dwelling pathogen *Giardia lamblia*. Journal of Immunology, 164, 1478–1487 10.4049/jimmunol.164.3.1478 10640765

[6] Feliziani C, Merino MC, Rivero MR, Hellman U, Pistoresi-Palencia MC, Rópolo AS. 2011. Immunodominant proteins α-1 giardin and β-giardin are expressed in both assemblages A and B of *Giardia lamblia*. BMC Microbiology, 11, 233 22011206 10.1186/1471-2180-11-233 PMC3206439

[7] Hanevik K, Kristofferson E, Svard S, Bruserud O, Ringqvist E, Sørnes S, Langeland N. 2011. Human cellular immune response against *Giardia lamblia* 5 years after acute giardiasis. Journal of Infectious Diseases, 204, 1779–1786 21990423 10.1093/infdis/jir639

[8] Hernández-Sánchez J, Liñan RF, M del R Salinas-Tobón, Ortega-Pierres G. 2008. *Giardia duodenalis*: Adhesion-deficient clones have reduced ability to establish infection in Mongolian gerbils. Experimental Parasitology, 119, 364–372 18456259 10.1016/j.exppara.2008.03.010

[9] Heyworth MF, Carlson JR, Ermak TH. 1987. Clearance of *Giardia muris* infection requires helper/inducer T lymphocytes. Journal of Experimental Medicine, 165, 1743–1748 2953846 10.1084/jem.165.6.1743 PMC2188375

[10] Heyworth MF, Vergara JA. 1994. *Giardia muris* trophozoite antigenic targets for mouse intestinal IgA antibody. Journal of Infectious Diseases, 169, 395–398 8106773 10.1093/infdis/169.2.395

[11] Jenikova G, Hruz P, Andersson MK, Tejman-Yarden N, Ferreira PCD, Andersen YS, Davids BJ, Gillin FD, Svärd SG, Curtiss R III, Eckmann L. 2011. α1-giardin based live heterologous vaccine protects against *Giardia lamblia* infection in a murine model. Vaccine, 29, 9529–9537 22001876 10.1016/j.vaccine.2011.09.126 PMC4045459

[12] Jenkins MC, O’Brien CN, Murphy C, Schwarz R, Miska K, Rosenthal B, Trout JM. 2009. Antibodies to the ventral disc protein δ-giardin prevent in vitro binding of *Giardia lamblia* trophozoites. Journal of Parasitology, 95, 895–899 19702359 10.1645/GE-1851R.1

[13] Kamda JD, Nash TE, Singer SM. 2012. *Giardia duodenalis*: Dendritic cell defects in IL-6 deficient mice contribute to susceptibility to intestinal infection. Experimental Parasitology, 130, 288–291 22248985 10.1016/j.exppara.2012.01.003 PMC3289762

[14] Langford TD, Housley MP, Boes M, Chen J, Kagnoff MF, Gillin FD, Eckmann L. 2002. Central importance of immunoglobulin A in host defense against *Giardia* spp. Infection and Immunity, 70, 11–18 11748158 10.1128/IAI.70.1.11-18.2002 PMC127595

[15] Lee P, Abdul-Wahid A, Faubert GM. 2009. Comparison of the local immune response against *Giardia lamblia* cyst wall protein 2 induced by recombinant *Lactococcus lactis* and *Streptococcus gordonii*. Microbes and Infection, 11, 20–28 18992359 10.1016/j.micinf.2008.10.002

[16] Li E, Zhao A, Shea-Donohue T, Singer SM. 2007. Mast cell-mediated changes in smooth muscle contractility during mouse giardiasis. Infection and Immunity, 75, 4514–4518 17620354 10.1128/IAI.00596-07 PMC1951189

[17] Nash TE. 1997. Antigenic variation in *Giardia lamblia* and the host’s immune response. Philosophical Transactions of the Royal Society of London B, 352, 1369–1375 10.1098/rstb.1997.0122 PMC1692022 9355129

[18] Nash TE. 2002. Surface antigenic variation in *Giardia lamblia*. Molecular Microbiology, 45, 585–590 12139606 10.1046/j.1365-2958.2002.03029.x

[19] O’Handley RM, Ceri H, Anette C, Olson ME. 2003. Passive immunity and serological immune response in dairy calves associated with natural *Giardia duodenalis* infections. Veterinary Parasitology, 113, 89–98 12695034 10.1016/s0304-4017(03)00059-1

[20] Olmez S, Aslan M, Yavuz A, Bulut G, Dulger AC. 2014. Diffuse nodular lymphoid hyperplasia of the small bowel associated with common variable immunodeficiency and giardiasis: a rare case report. Wiener klinische Wochenschrift, 126, 294–297 24647448 10.1007/s00508-014-0525-5

[21] Palm JED, Weiland ME-L, Griffiths WJ, Ljungström I, Svärd SG. 2003. Identification of immunoreactive proteins during acute human giardiasis. Journal of Infectious Diseases, 187, 1849–1859 12792861 10.1086/375356

[22] Peattie DA, Alonso RA, Hein A, Caulfield JP. 1989. Ultrastructural localization of giardins to the edges of disk microribbons of *Giardia lamblia* and the nucleotide and deduced protein sequence of alpha giardin. Journal of Cell Biology, 109, 2323–2335 2808530 10.1083/jcb.109.5.2323 PMC2115877

[23] Priest JW, Moss DM, Visvesvara GS, Jones CC, Li A, Isaac-Renton JL. 2010. Multiplex assay detection of immunoglobulin G antibodies that recognize *Giardia intestinalis* and *Cryptosporidium parvum* antigens. Clinical and Vaccine Immunology, 17, 1695–1707 20876825 10.1128/CVI.00160-10 PMC2976096

[24] Rivero FD, Saura A, Prucca CG, Carranza PG, Torri A, Lujan HD. 2010. Disruption of antigenic variation is crucial for effective parasite vaccine. Nature Medicine, 16, 551–557 10.1038/nm.2141 20418884

[25] Schofield PJ, Edwards MR, Matthews J, Wilson JR. 1992. The pathway of arginine catabolism in *Giardia intestinalis*. Molecular and Biochemical Parasitology, 51, 29–36 1314332 10.1016/0166-6851(92)90197-r

[26] Singer SM, Elmendorf HG, Conrad JT, Nash TE. 2001. Biological selection of variant-specific surface proteins in *Giardia lamblia*. Journal of Infectious Diseases, 183, 119–124 11087204 10.1086/317659

[27] Singer SM, Nash TE. 2000. T-cell-dependent control of acute *Giardia lamblia* infections in mice. Infection and Immunity, 68, 170–175 10603384 10.1128/iai.68.1.170-175.2000 PMC97117

[28] Smith PD, Gillin FD, Brown WR, Nash TE. 1981. IgG antibody to *Giardia lamblia* detected by enzyme-linked immunosorbent assay. Gastroenterology, 80, 1476–1480 7014351

[29] Stadelmann B, Hanevik K, Andersson MK, Bruserud O, Svärd SG. 2013. The role of arginine and arginine-metabolizing enzymes during *Giardia*-host cell interactions *in vitro*. BMC Microbiology, 13, 256 24228819 10.1186/1471-2180-13-256 PMC4225669

[30] Stadelmann B, Merino MC, Persson L, Svärd SG. 2012. Arginine consumption by the intestinal parasite *Giardia intestinalis* reduces proliferation of intestinal epithelial cells. PLoS ONE, 7(9), e45325 23028934 10.1371/journal.pone.0045325 PMC3446895

[31] Tako EA, Hassimi MF, Li E, Singer SM. 2013. Transcriptomic analysis of the host response to *Giardia duodenalis* infection reveals redundant mechanisms for parasite control. mBio, 4(6), e00660-13 24194537 10.1128/mBio.00660-13 PMC3892777

[32] Téllez A, Palm D, Weiland M, Alemán J, Winiecka-Krusnell J, Linder E, Svärd S. 2005. Secretory antibodies against *Giardia intestinalis* in lactating Nicaraguan women. Parasite Immunology, 27, 163–169 15987339 10.1111/j.1365-3024.2005.00758.x

[33] Weiland ME-L, McArthur AG, Morrison HG, Sogin ML, Svärd SG. 2005. Annexin-like alpha giardins: a new cytoskeletal gene family in *Giardia lamblia*. International Journal for Parasitology, 35, 617–626 15862575 10.1016/j.ijpara.2004.12.009

[34] Weiland ME-L, Palm JED, Griffiths WJ, McCaffery JM, Svärd SG. 2003. Characterisation of alpha-1 giardin: an immunodominant *Giardia lamblia* annexin with glycosaminoglycan-binding activity. International Journal for Parasitology, 33, 1341–1351 14527517 10.1016/s0020-7519(03)00201-7

[35] Wenman WM, Meuser RU, Nyugen Q, Kilani RT, El-Shewy K, Sherburne R. 1993. Characterization of an immunodominant *Giardia lamblia* protein antigen related to alpha giardin. Parasitology Research, 79, 587–592 8278341 10.1007/BF00932243

[36] Zhou P, Li E, Zhu N, Robertson J, Nash T, Singer SM. 2003. Role of interleukin-6 in the control of acute and chronic *Giardia lamblia* infections in mice. Infection and Immunity, 71, 1566–1568 12595478 10.1128/IAI.71.3.1566-1568.2003 PMC148826

